# pH-Responsive Gamma-Irradiated Poly(Acrylic Acid)-Cellulose-Nanocrystal-Reinforced Hydrogels

**DOI:** 10.3390/polym12091932

**Published:** 2020-08-27

**Authors:** Wan Hafizi Wan Ishak, Oo Yong Jia, Ishak Ahmad

**Affiliations:** 1 Department of Chemical Sciences, Faculty of Science and Technology, Universiti Kebangsaan Malaysia, Selangor 43600, Malaysia; hafizi.wan29@gmail.com (W.H.W.I.); oo_yj93@hotmail.com (O.Y.J.); 2 Polymer Research Center (PORCE), Faculty of Science and Technology, Universiti Kebangsaan Malaysia, Selangor 43600, Malaysia

**Keywords:** hydrogel, pH sensitive, cellulose nanocrystal, radiation crosslinking, stimuli response

## Abstract

A pH-sensitive poly(acrylic acid) composite hydrogel was successfully synthesized via gamma irradiation and reinforced with cellulosic materials of different sizes. Cellulose was extracted from rice husks via alkali and bleaching treatment, and an acid hydrolysis treatment was performed to extract cellulose nanocrystals (CNCs). Morphological observation of cellulose and CNCs using scanning electron microscopy (SEM) and transmission electron microscopy (TEM) revealed diameters of 22–123 μm and 5–16 nm, respectively. The swelling properties of the fabricated poly(acrylic acid)/cellulosic hydrogels were found to respond to changes in pH, and CNC-reinforced hydrogels performed better than cellulose-reinforced hydrogels. The highly crystalline CNC provided a greater storage modulus, hence acting as a better reinforcing material for poly(acrylic acid)-based hydrogels. SEM showed that hydrogels reinforced with the CNC nanofillers contained a homogeneous pore distribution and produced better interfacial interactions than those reinforced with the cellulose microfillers, thus performing better as hydrogels. These findings demonstrate that gamma-irradiated poly(acrylic acid) hydrogels reinforced with CNCs exhibit a better stimuli response toward pH than poly(acrylic acid) hydrogels reinforced with cellulose.

## 1. Introduction

Recently, hydrogels that can inherently display dramatic changes in response to environmental stimuli such as temperature, pH, and certain chemicals have generated considerable research interest [1,2]. Furthermore, hydrogels that simultaneously respond to several are desirable as more potential applications emerge [3]. Current research is focused pH-sensitive hydrogels, as the parameter are fundamental in biomedical systems [4,5,6]. The development of unique stimuli-responsive hydrogels is therefore needed for applications in numerous biomedical fields, including drug delivery, artificial organ development, wound dressing, and tissue engineering [1,2,7].

Owing to the presence of a carbon–carbon double bond (C=C) in acrylic acid (AA), its reactivity is very high [8], and it can react easily with free radicals and crosslink through a radical polymerization process to produce poly(acrylic acid) (PAA) hydrogels. With the presence of carboxylic acid groups (COOH) in the PAA chains, the swelling ability largely depends on the surrounding pH, offering practical pharmaceutical applications, such as drug delivery [9]. For example, the pKa for AA is between 4.5 and 5.0; more swelling will occur at pH > 5, such as in the small intestine, whereas at pH < 4, such as in the stomach, PAA will only swell to a small extent [10]. As the term “network” implies, crosslinks must form to avoid dissolving or losing the structural integrity of the hydrogel in the aqueous phase [11]. There are various techniques to prepare hydrogels, which can be divided into three major groups: chemical crosslinking, physical crosslinking, and radiation crosslinking [12,13,14].

Conventional chemical crosslinking improves mechanical and thermal stability, although the use of chemical crosslinking to induce crosslinking leads to concerns about toxicity, as well as a limited swelling capability and slow stimuli response [15,16]. Physical crosslinking provides physical interactions through hydrophobic interactions or hydrogen bonding, which are non-toxic reactions. However, these hydrogels are not sufficiently stable due to weak interactions that are inappropriate for effective drug-delivery systems [17]. As an alternative, gamma radiation offers some advantages to induce crosslinking, as it does not require any additional chemical agents, and crosslinking is easily controlled by altering the radiation dose [18,19]. Therefore, this method not only avoids the use of additional chemicals but can also maintain the biocompatibility of the polymer. When the crosslinking prevails, the mechanical properties of PAA is improved. However, the fabrication of hydrogels using irradiation presents a challenge because a high dose of gamma radiation may generate excess free-radicals that are potentially harmful and unsuitable for medical applications [20]. Meanwhile, to overcome weaknesses related to mechanical properties, synthetic polymers can be reinforced with natural fibers to improve properties such as swelling and toughness [21].

Cellulose nanocrystals (CNCs) are promising biopolymers used as reinforcing materials for their superior mechanical properties, high biocompatibility, hydrophilicity, and low density [5]. The extraction and modification of CNCs from natural fibers such as rice husk, kenaf, and empty fruit bunch (EFB) have been extensively studied to obtain the benefits of CNCs [22,23]. However, alkali and bleaching treatments are necessary to extract the cellulose by removing lignin and hemicellulose, while an acid hydrolysis treatment is subsequently performed to produce CNCs [22,23]. Previously, Lim et al. [5] added CNCs to PAA hydrogels using a chemical crosslinking method, and their results showed some improvements in the swelling and mechanical properties of hydrogels. In a similar study on the use of CNC in PAA hydrogels, Li et al. [24] found that the elastic modulus, breaking strength, and elongation at break of the nanocomposite hydrogels were significantly higher than those of the PAA hydrogel without CNC. Nevertheless, the use of chemical additives as crosslinkers was unavoidable in both studies. Although many studies have used CNCs as reinforcing materials in polymer hydrogels, no research has been reported on the effects of gamma-irradiated PAA/CNC hydrogels.

This study aimed to prepare a hydrogel with favorable properties by gamma irradiation of the PAA composites reinforced with either cellulose or CNCs. The samples were characterized using Fourier-transform infrared (FTIR) spectroscopy, transmission electron microscopy (TEM), and scanning electron microscopy (SEM). The effect of the filler size (i.e., nano- or microsized) on the gel fraction, swelling behavior, and elastic properties of the hydrogels was also investigated.

## 2. Materials and Methods

### 2.1. Materials

Rice husk for use as raw fibers was supplied byPadiberas Nasional Berhad (BERNAS), Shah Alam, Selangor, Malaysia. Glacial acetic acid, acrylic acid, and sulfuric acid were purchased from Sigma-Aldrich (M) (Petaling Jaya, Selangor, Malaysia). Sodium hydroxide and sodium chlorite were purchased from SYSTERM-chemAR (Shah Alam, Selangor, Malaysia).

### 2.2. Preparation of Cellulose and Cellulose Nanocrystals (CNCs)

Rice husk fibers were ground to a powder, which was subsequently washed with distilled water before undergoing chemical treatment. The powder was treated with 4% sodium hydroxide (NaOH) aqueous solution under reflux at 90 °C for 3 h; this process was performed in triplicate. This alkali-treated powder was then bleached with a 1.7% sodium chlorite (NaClO_2_) aqueous solution at pH 4.5 under reflux at 100 °C for 4 h, as described by Ooi et al. [23] in order to extract the cellulose. A mechanical stirrer was used during both processes. After each treatment, the fibers were washed with distilled water and dried at 25 °C.

Subsequently, acid hydrolysis treatment were performed in order to prepare CNCs. Under controlled condition, 15 g of cellulose powder were continuously stirred in 60% sulphuric acid (H_2_SO_4_) solution. A previously published procedure was followed to prepare the CNCs [23]. The CNC suspension was transferred to an ice bath to stop the reaction and then washed with distilled water via centrifugation several times at 4500 rpm and 10 °C for 10 min to remove the acid. The CNC suspension was then dialyzed for several days until its pH became neutral. It was then stored in a refrigerator and finally freeze-dried to obtain the CNC powder.

### 2.3. Preparation of Hydrogels

**Figure 1:**
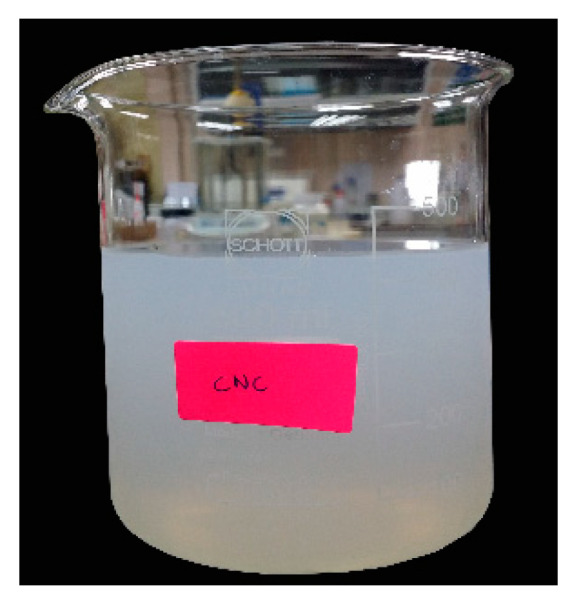
AA mixed with the stable redispersed freeze-dried CNC suspension.

AA was mixed with distilled water and either cellulose or CNCs to produce hydrogels via gamma radiation. The PAA hydrogel fabrication reinforced with 4% or 8% cellulose is briefly outlined as follows. Cellulose (0.20 g) was mixed with distilled water, ultrasonicated (Bransonic CPXH, Branson Ultrasonic, Danbury, CT, USA) for 50 min at 25 °C and homogenized (IKA T-25 homogenizer, IKA-Werke, GmbH, Staufen im Breisgau, Germany) for 30 min at 8000 rpm to ensure a uniform suspension. The cellulose suspension was then heated to 50 °C before adding an AA (4.8 g) solution and stirring with a magnetic stirrer for 30 min. The solution was then transferred to a petri dish and finally exposed to gamma irradiation (Gamma Cell 220 Excel, MDS Nordion, Ottawa, ON, Canada) at a dose of 30 kGy (dose rate: 1.5 kGy/hour, 25 °C) to obtain the hydrogel; this dose was selected based on previous studies [25,26]. PAA hydrogels without cellulose or CNC (control) and PAA hydrogels reinforced with 4% or 8% CNCs were prepared following the same procedure. The various compositions of the hydrogels produced are shown in Table 1, and Figure 1 shows the AA mixed with redispersed freeze-dried CNC stable suspension before exposure to gamma irradiation.

### 2.4. Morphological Observation

TEM was used to determine the dimensions and morphology of the CNCs. The CNC suspension was ultrasonicated for 50 min to disperse the agglomerated particles. One drop of diluted suspension (1 wt %) was deposited on the surface of the copper grid and coated with a thin carbon film. It was then negatively stained with 2% uranyl acetate solution for 5 min and allowed to dry at room temperature. The sample was observed at 35,000× magnification using a Philips CM30 microscope (North Billerica, MA, USA) operated at 100 kV accelerating voltage. Cellulose was placed on an Al stub and incubated in an oven at 60 °C, and then the surface morphology of the thus-obtained samples was observed at 250× magnification and 15 kV accelerating voltage with SEM (Philips XL 30, North Billerica, MA, USA).

SEM (Philips XL 30) was also used to observe the morphology of the hydrogel cross-sections. The samples were first immersed in distilled water for 2 d to allow the hydrogels to swell. The samples (diameter = 10 mm, thickness = 5 mm) were then freeze-dried at −50 °C for 36 h and coated with gold (0.01–0.1 μm thick). The morphology was then observed at 200× magnification and 15 kV accelerating voltage.

### 2.5. Fourier-Transform Infrared (FTIR) Spectroscopy

FTIR spectroscopy (2000 Perkin Elmer instrument (Hopkinton, MA, USA)) was used to identify the functional groups present in the samples. All samples were dried before subjected to FTIR. Four samples were tested, which were the original rice husk and the rice husk after each treatments. In addition, monomer AA, PAA hydrogels, PAA/8% cellulose hydrogels, and PAA/8% CNC hydrogels were also examined. FTIR spectra were recorded from 4000 cm^−^^1^ to 400 cm^−^^1^.

### 2.6. Gel Fraction Test

Gel fraction analysis was performed to determine the gelation percentage for each hydrogel composition. The samples were dried in an oven at 37 °C until a constant weight was obtained (W_1_). The dried samples were then soaked in distilled water at room temperature for three days in order to remove any extra soluble material. The samples were then removed from the distilled water and dried again in an oven at 37 °C until there was no change in weight (W_2_). The gel fraction (GF) could then be calculated from Equation (1):

(1) $$\mathrm{GF}\;\left(\%\right)=\frac{W_{2}}{W_{1}}\times 100$$

### 2.7. Swelling Test

A swelling test was performed in order to investigate the swelling properties of hydrogels toward different temperature and pH response. First, the hydrogel samples were dried in an oven to obtain a constant weight (W_i_). Next, they were separately immersed in 50 mL of distilled water (pH 6.7) at 25 °C and 37 °C for 72 h to evaluate the effect of temperature or in 50 mL of buffer solution at pH 3, 7, and 11 to investigate the response to pH. The samples were then removed from the solutions at certain time intervals and weighed again as swollen samples (W_f_). The swelling degree (SD) could be calculated using Equation (2):

(2) $$\mathrm{SD}\;\left(\%\right)=\frac{W_{f}-W_{i}}{W_{i}}\times 100$$

### 2.8. Rheology Test

Rheology measurements were performed using an Anton Paar Physica MRC 301 instrument (Anton Paar GmbH., Ostfildern, Germany) to determine the influence of the AA/filler ratio on the elastic properties of the hydrogels. All samples were cut into discs (diameter = 10 mm, thickness = 5 mm), and the storage modulus was recorded with 0.05% shear strain at 25 °C, a 1.0 mm parallel plate gap, and an angular frequency from 0.1 to 100 Hz.

## 3. Results

### 3.1. Morphological Observation of Cellulose and Cellulose Nanocrystals

**Figure 2:**
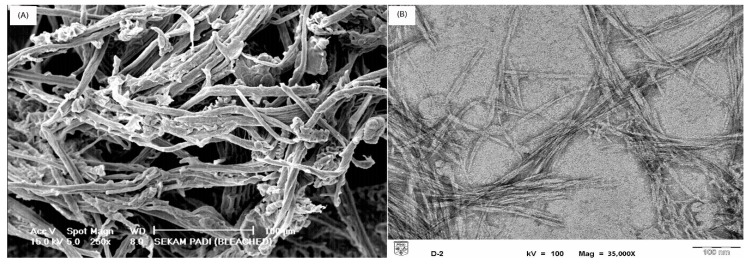
(**A**) Scanning electron microscope (SEM) image of pristine cellulose fibers and (**B**) transmission electron microscope (TEM) image of CNCs.

Figure 2 shows the SEM micrograph of cellulose from rice husk after bleaching and the TEM micrograph of CNCs obtained from the acid hydrolysis of cellulose. The diameter of cellulose fibers is 22–123 μm (Figure 2a), whereas the diameter of CNCs is significantly smaller (5–16 nm; Figure 2b); acid hydrolysis treatment of cellulose fibers with diameters in the micrometer range affords CNCs as a reinforcing material with diameters in the nanometer range.

The needle-like structure of the CNCs is clearly observed in Figure 2b, which is due to the cleavage of the amorphous region upon acid hydrolysis while leaving the crystalline region intact [23]. The obtained CNCs with aspect ratios of 10–21, in conjunction with their known desirable mechanical properties, are expected to serve as an excellent reinforcing material [27]. The use of sulfuric acid in hydrolysis treatments is well known to provide isolated crystalline fragments, and it produces a negative electrostatic layer that stabilizes the suspension, thus providing a better dispersion [28]. The incorporation of CNCs into hydrogels is thus expected to provide stronger gels while improving pore formation.

### 3.2. Fourier-Transform Infrared (FTIR) Spectroscopy

**Figure 3:**
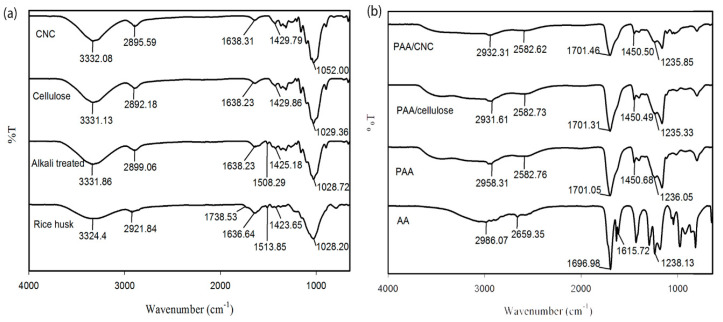
Fourier-transform infrared (FTIR) spectra of (**a**) raw rice husk fibers, alkali-treated rice husk fibers, cellulose, and CNCs; (**b**) monomer AA, pure poly(acrylic acid) (PAA) hydrogels, PAA/cellulose hydrogels, and PAA/CNC hydrogels.

Figure 3 shows the FTIR spectra for the raw fibers and the fibers after each chemical treatment, as well as the AA monomer and PAA hydrogels after polymerization. Some important absorption peaks have been selected in order to identify changes in the chemical structure among the samples. In Figure 3a, the broad peak from 3333 cm^−1^ to 3324 cm^−1^ corresponds to the O–H stretching of intermolecular hydrogen bonding, while the peak at 1638 cm-1 is attributed to O–H bending. The peaks around 2895 cm^−1^, 1429 cm^−1^, and 1052 cm^−^^1^ are attributed to C–H stretching, symmetry CH_2_ bending, and C–O stretching of glucopyranic rings, respectively. These peaks therefore confirm that the cellulose component of the rice husk is not removed during the chemical treatment processes [23].

However, the disappearance of the peak at 1738 cm^−1^ in the FTIR spectrum of the alkali-treated rice husk indicates the removal of the C=O bonds from the carboxylic acid and ester groups of hemicellulose, indicating that the alkaline treatment successfully removes hemicellulose [23,28]. The intensity of the peak at 1510 cm^−1^, associated with the C=C stretching of the aromatic ring of lignin, decreases after the first treatment and completely disappears after the second treatment, indicating the removal of lignin during bleaching [29]. The FTIR spectrum of the CNCs is nearly the same as that of cellulose, demonstrating that the chemical structure does not change upon acid hydrolysis [23,28].

The peaks at 1701 cm^−1^ and 1235 cm^−1^ in Figure 3b correspond to the C=O stretching and the C–O stretching of the carboxylate groups of the monomer AA, respectively. The broad peak from 3350 cm^−1^ to 2750 cm^−1^ is attributed to the OH group. The FTIR spectrum of the AA monomer exhibits a distinct peak at 1615 cm^−1^, which corresponds to C=C stretching. In the PAA FTIR spectrum, this peak is absent, and a new peak attributed to C–C bonds emerges at 1450 cm^−1^, revealing that the C=C group has been transformed to a C–C covalent bond and that successful crosslinking has occurred during the process of hydrogel formation [30].

**Figure 4:**
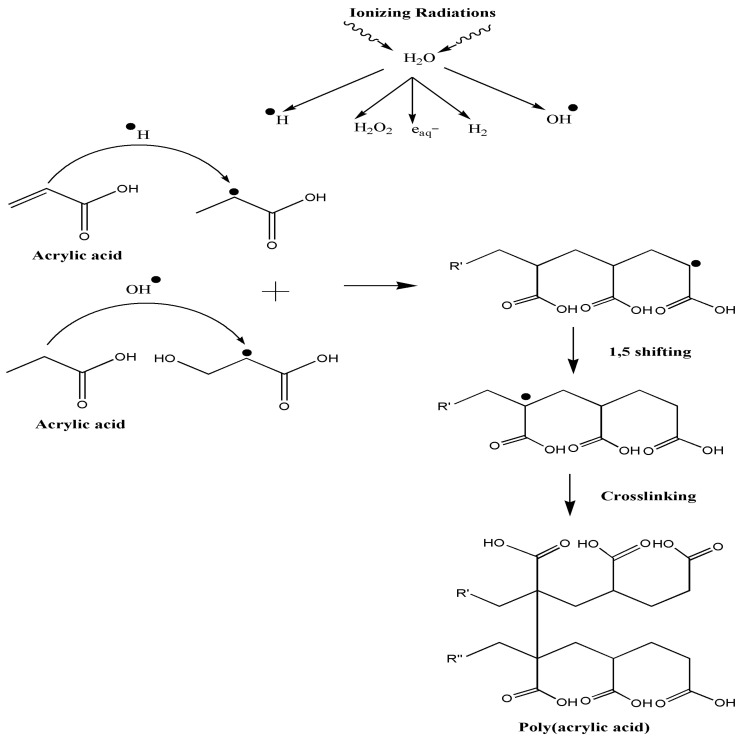
Reaction mechanism for PAA hydrogel synthesis [30].

During crosslinking, gamma rays produce free radicals and convert the C=C bonds into C–C bonds [30]. A hydrogel forms via the irradiation of the aqueous polymer solution owing to reactions between free radicals produced by water hydrolysis and the active sites of the polymers [18]. High-energy gamma radiation induces the water molecules to produce products such as hydrogen free radicals (H•), hydroxide free radicals (OH•), and electrons (e-) as the nucleophiles. Both H• and OH• attack the active sites of C=C in the AA monomers and produce a long straight chain of PAA free radicals (PAA•) while forming C–C covalent bonds. Finally, the long straight PAA• chains crosslink intermolecularly and form a three-dimensional (3D) PAA hydrogel structure. For the PAA hydrogel spectra reinforced with each type of filler (i.e., cellulose or CNCs), the absorption peaks are the same as those for the unreinforced PAA hydrogel, suggesting that the formation of both reinforced hydrogels involves the same reaction between the fillers and the polymer chains in the matrix, without further affecting the polymer structure. This finding demonstrates that the resulting free radicals only affect the AA (to produce PAA), but do not alter the fillers. The reaction mechanisms are shown in Figure 4.

The FTIR spectra for both PAA/cellulose and PAA/CNC hydrogels do not significant differ from that of the PAA hydrogel, which suggests that crosslinking only occurs between the AA monomers without affecting any other functional groups of the cellulose or CNC backbones. It therefore appears that the cellulosic material is entrapped within the PAA 3D matrix, thus forming semi-interpenetrating networks (semi-IPNs) of PAA/cellulose or PAA/CNC hydrogels. There is no characteristic peak of cellulosic material visible in the FTIR spectra for both PAA/cellulose and PAA/CNC hydrogels due to low concentration of fillers. Previously, Lim et al. [5] reported the formation of semi-IPN PAA/CNC hydrogels with the presence of C–O groups attributed to CNC characteristic in the FTIR spectrum of PAA/CNC hydrogels as higher concentration of CNC (25%) was reinforced.

### 3.3. Gel Fraction Test

**Figure 5:**
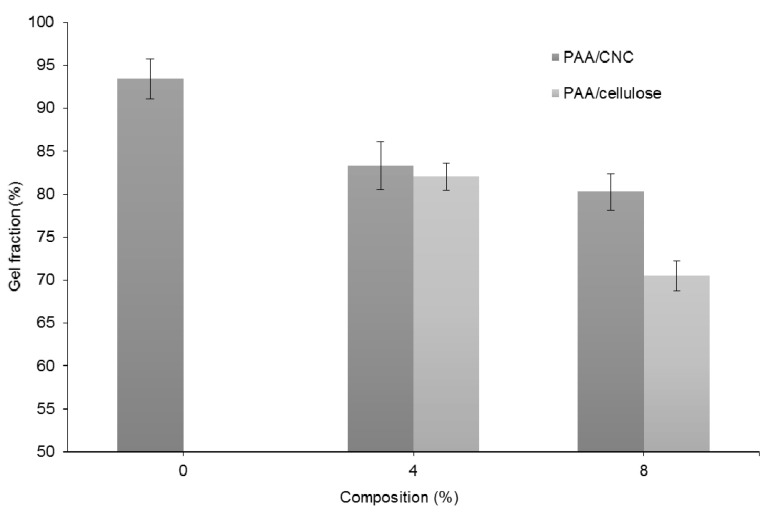
Effect of cellulose and CNC composition on the gel fraction of PAA hydrogels.

Figure 5 shows how the gel fraction varies with the cellulose and CNC composition in the hydrogels. Importantly, the gel fraction of the hydrogels decreases as the content of added cellulose or CNCs increases. The high concentration of AA in pure PAA results in a higher crosslinking density of PAA, as crosslinking by gamma radiation successfully transforms the linear polymer into a 3D network that reduces the solubility of organic solvents [31]. Therefore, the addition of cellulosic or CNC material reduces the concentration of AA, thus lowering the crosslinking density and the resulting gel fraction.

Figure 5 reveals that the gel fractions of the hydrogels containing PAA/CNC are higher than those of PAA/cellulose hydrogels. Since the PAA/CNC and PAA/cellulose hydrogels produce a semi-IPN, hydrogen bonds form between the PAA and either cellulose or CNC chemical backbones, thus introducing intermolecular bonds between the molecules. Therefore, the nanosized CNCs offer a better dispersion and larger surface area than microsized cellulose for hydrogen bonding interactions with PAA molecules.

### 3.4. Swelling Test

#### 3.4.1. Effect of Different Temperature

**Figure 6:**
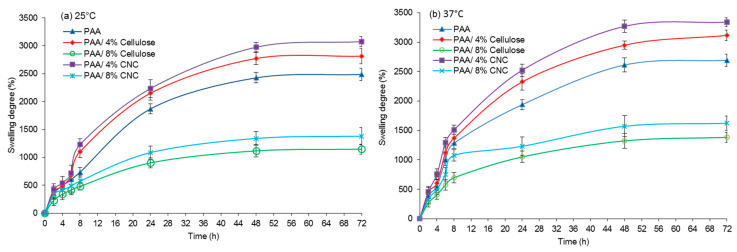
Extent of hydrogel swelling at (**a**) 25 °C and (**b**) 37 °C.

One important parameter for evaluating the properties of hydrogels is their capability to swell in water without the solvation of polymeric content. Figure 6 shows the swelling behavior of both reinforced hydrogels (PAA/cellulose and PAA/CNC) and the filler-free PAA hydrogel used as a control at 25 °C and 37 °C. Generally, all samples exhibited an increase in the extent of swelling over time, which justifies their ability to act as hydrogels [32]. The incorporation of cellulosic materials (cellulose and CNCs) as reinforcements modifies the water uptake speed and absorption ability of the hydrogels.

The hydrophilic properties of cellulose, which are due to the presence of OH groups, attract water molecules, thus increasing the extent of swelling [5]. Both 4%-reinforced PAA hydrogels (with 4% cellulose or CNCs as the reinforcements) absorbed more water than PAA hydrogels without fillers. During water absorption, the matrix hydrogel swells and pores open to enable further penetration of water. The diffusion of water molecules into the polymer chain induces the formation of a rubbery (swollen) region by facilitating the relaxation of the polymer network [33]. Both cellulose and CNCs act as supporting systems, stabilizing the hydrogel pores during polymer network relaxation.

However, the size of the reinforcement filler plays a key role in determining the amount of water uptake and absorption speed. Reinforced PAA/CNC hydrogels, which contain nanosized CNCs, exhibited a greater degree of swelling than hydrogels reinforced with microsized cellulose. The nanosize of CNCs allows a better dispersion, thus facilitating the formation of more rigid and stable pores while also providing greater interstitial volume and surface area [33]. Based on Figure 6, it can be conclude that the 4% filler composition produces the best hydrogel swelling. Conversely, PAA hydrogels reinforced with 8% CNC or 8% cellulose produce less swelling than pure PAA hydrogel alone. This phenomenon can be ascribed to an excess of reinforcements, which causes the formation of a compact matrix, thus introducing difficulties for polymer relaxation and subsequently reducing the water absorption speed and uptake.

**Figure 7:**
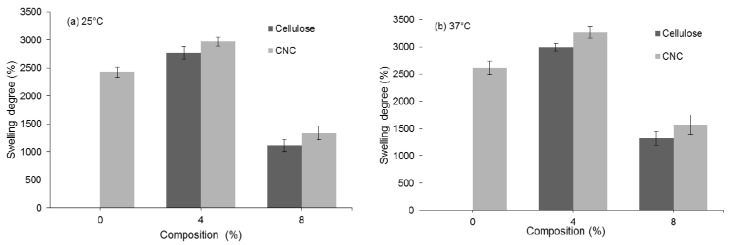
Swelling degree of PAA/cellulose and PAA/CNC hydrogels after 24 h incubation at (**a**) 25 °C and (**b**) 37 °C.

With regard to the different temperatures (Figure 7), the swelling behavior of the hydrogels with all compositions was compared at (a) 25 °C and (b) 37 °C, and the swelling capability was found to be better at higher temperature (37 °C). PAA hydrogels in water dissociated as the temperature increased due to the simultaneous hydration of the polymer with increasing temperature [34]. In the semi-IPN PAA hydrogels, this effect should be stronger owing to the higher density of associations between the PAA groups, which should result in larger swelling with increasing temperature [5]. Higher temperature induces the pores to swell faster and become larger, thus facilitating the penetration of water into the hydrogels. Therefore, water is absorbed faster and to a greater extent, resulting in increased swelling. This result successfully demonstrates the swelling capability of the fabricated hydrogels in different temperature, which is promising for use in biomedical and pharmaceutical applications.

#### 3.4.2. pH Response

**Figure 8:**
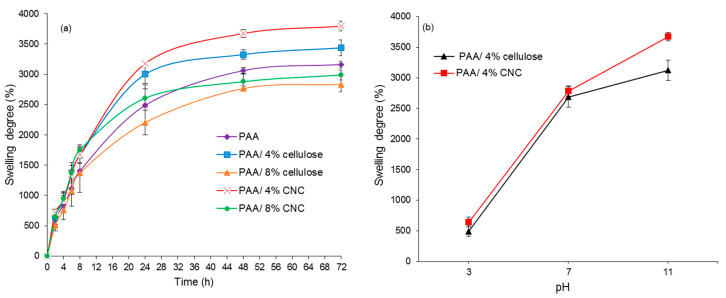
(**a**) Effect of filler composition on the swelling percentage of PAA hydrogels at pH 11 and (**b**) effect of pH on swelling percentage of PAA hydrogels.

The influence of swelling time related to cellulose and CNC composition, as expressed as swelling percentage for all hydrogels, was investigated (Figure 8a). The trend in the swelling degree is the same for all pH studied (pH 3, 7, and 11); only the swelling performance at pH 11 is shown. Similar to Figure 6, the swelling degree performance is the same for all samples at pH 11. The highest swelling degree is obtained by PAA hydrogels reinforced with 4% CNC (or 4% cellulose), followed by pure PAA hydrogels and PAA hydrogels reinforced with 8% CNC (or 8% cellulose). Again, the size of reinforced cellulosic material is the most dominant factor to determine the swelling capability of prepared hydrogels despite different pH environments [19]. Therefore, the extent of swelling can be controlled by altering the size and amount of filler used (i.e., filler type and % filler).

The variations in the swelling percentage for hydrogels at pH 3, 7, and 11 are shown in Figure 8b, which demonstrate that the PAA hydrogels are responsive to pH. The swelling percentage increases significantly from pH 3 to pH 7 and further increases but to a lesser extent at pH 11. PAA is a polyelectrolyte because it contains the functional group COOH, with a pKa of 4.25 [35]; the swelling percentage of these PAA hydrogels is low at acidic pH owing to unsuitable conditions for the carboxylic acid (COOH) group to ionize to the negatively charged carboxylate ion (COO^−^). When the pH exceeds 4.25, the swelling percentage increases as the COOH groups begin to ionize and form COO^−^ anions, as shown in the Equation (3) [35]. As the pH increases and deviates farther from the pKa value of PAA, a stronger driving force is created by the ionization of the carboxylate groups. In turn, greater electrostatic repulsion occurs between the ionic chains, thus resulting in greater free volume space and an increased swelling ratio [36].

(3) $$\mathrm{RCOOH}+{\mathrm{OH}}^{-}↔{\;\mathrm{RCOO}}^{-}+H_{2}O$$

### 3.5. Scanning Electron Microscopy (SEM)

**Figure 9:**
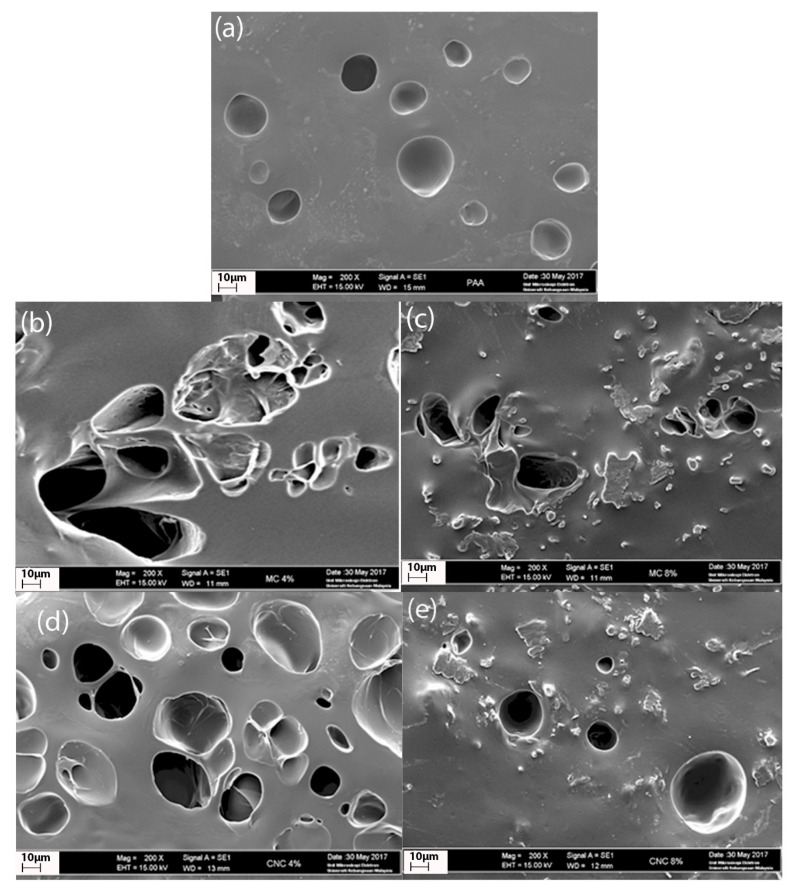
SEM micrographs of (**a**) PAA hydrogels, (**b**) PAA/4% cellulose hydrogels, (**c**) PAA/8% cellulose hydrogels, (**d**) PAA/4% CNC hydrogels, and (**e**) PAA/8% CNC hydrogels.

Figure 9 shows SEM images of all of the prepared hydrogels to distinguish the pore differences. With the presence of fillers, the formation of 3D porous structures can be observed. The distribution of pores in the PAA/CNC hydrogels is more even and homogeneous throughout the surface (Figure 9d,e) than that in the PAA/cellulose hydrogels (Figure 9b,c), and hence, the swelling degree of both hydrogels is affected. The homogeneous pores in PAA/CNC hydrogels are due to the well-dispersed CNC in the PAA matrix, which improves the formation and distribution of pores. Notably, the sulfuric acid hydrolysis treatment provides nanosized isolated fragments (CNC) and creates an electrostatic layer of sulfate groups on the cellulosic surface, thus forming a CNC suspension with a better, more stable dispersion [28].

A uniform, homogeneous distribution of hydrogel pore structures is expected to allow water molecules to absorb in all directions, thus increasing the swelling percentage. The PAA/CNC hydrogels exhibit a smoother surface than the PAA/cellulose hydrogels, which indicates that if the fillers are smaller, the interfacial interactions will be better owing to the more homogeneous dispersion of fillers within the polymer matrix. However, the excess amount of fillers in the PAA hydrogels with both 8% cellulose and CNC leads to the congestion of pores, as presented in Figure 9c,e, thus reducing the water intake capacity.

### 3.6. Rheology Test

**Figure 10:**
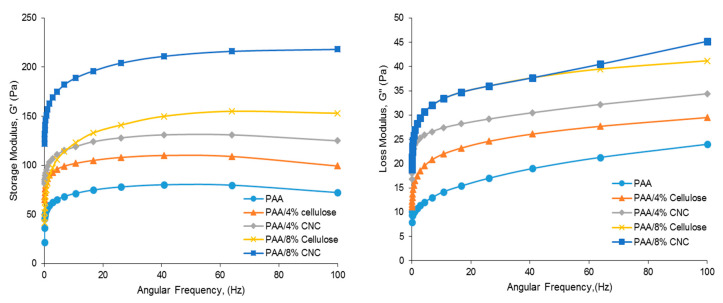
Effect of filler composition on the storage (**left**) and loss (**right**) modulus of PAA hydrogels.

The storage modulus (G′) is typically defined as a measurement of the energy stored in a material and represents the elastic portion of its flow behavior while the loss modulus (G″) is a measurement of viscosity of a solid material. Elastic materials can immediately change their shape when a shear force is imposed upon them and return to their original shape when the shear force is released. For both the cellulose and CNC-filler-reinforced hydrogels, G′ and G″ increases significantly, as shown in Figure 10. Furthermore, the G′ and G″ of both types of hydrogel further increases with 8% filler. This trend was previously observed with increasing filler composition [37]. As both the polymer chains and fillers are hydrophilic, the fillers can homogeneously disperse within the hydrogel matrices, resulting in the more effective transfer of stress from the polymer chains to the filler and inhibiting cracks in the hydrogels. Furthermore, all hydrogels exhibit the G′ value of more than 50 Pa while the G″ is less than 50 Pa. Since the value of G′ > G″, these hydrogels demonstrate viscoelastic properties.

Figure 10 also clearly reveals that PAA/CNC hydrogels exhibit a higher G′ and G″ than the PAA/cellulose hydrogels. When the amorphous area in the cellulose content was dissolved during the chemical treatment, the crystalline structure with a higher average aspect ratio remains and indirectly improves adhesion between the polymer chains and filler [38]. Because the nanocrystalline CNCs more effectively strengthen the hydrogel matrix, the rigidity of the PAA/CNC hydrogels is thus increased [39]. This finding is consistent with those of a previous study, wherein the the highly crystalline CNCs were observed to considerably strengthen composite materials [27]. In addition, during acid hydrolysis, more active hydroxyl groups are provided on the surface of the CNCs, thus allowing more interfacial interactions to occur between the PAA and CNCs.

## 4. Conclusions

The modification of cellulose fibers loaded during hydrogel production affects the hydrogel performance. As the size of cellulose fibers is reduced (from cellulose to CNCs), the swelling and rigidity of the resulting PAA hydrogels increases owing to the formation of more pores and interfacial interactions. The above results demonstrate that hydrogels reinforced with nanosized CNCs from the acid hydrolysis of cellulose result in improved hydrogel performance and greater potential as biocomposite materials. Significantly, PAA/CNC hydrogels also show a remarkable response toward pH, thus suggesting their potential as a drug-delivery system.

## Figures and Tables

**Table 1:** Compositions of hydrogels.

Composition	Cellulose/CNC (g)	Water (mL)	Acrylic Acid (AA) (g)
0%	0.00	30.00	5.00
4%	0.20	30.00	4.80
8%	0.40	30.00	4.60
